# Physical activity and sedentary behavior in Belgium (BNFCS2014): design, methods and expected outcomes

**DOI:** 10.1186/s13690-016-0156-6

**Published:** 2016-10-17

**Authors:** Thérésa Lebacq, Cloë Ost, Sarah Bel, Loes Brocatus, Eveline Teppers, Koenraad Cuypers, Jean Tafforeau, Karin A. A. De Ridder

**Affiliations:** Scientific Institute of Public Health, Department of Public Health and Surveillance, Unit Surveys, Lifestyle and Chronic Diseases, Brussels, Belgium

**Keywords:** Accelerometer, Physical activity, Sedentary behavior, Self-reported questionnaire

## Abstract

**Background:**

There is strong evidence to indicate that regular moderate intensity physical activity is associated with health benefits. Furthermore, sedentary behavior has been related with an increased risk for all-cause mortality. The accurate measurement of physical activity and sedentary behavior is therefore vital to evaluate their health impact and provide evidence for the development of public health recommendations. This paper describes the methodology used for assessing physical activity and sedentary behavior in the Belgian population in the context of the Belgian National Food Consumption Survey 2014 (BNFCS2014).

**Results:**

Data about physical activity and sedentary behavior were collected as part of the cross-sectional BNFCS2014 between February 2014 and May 2015. A nationally-representative sample of children (3–9 years) and adolescents (10–17 years) were asked to wear an accelerometer (Actigraph® GT3X) during their waking hours for 7 consecutive days. Data were recorded in 15-second epochs and respondents with at least 2 valid week days (i.e., 10 h of wear-time) and 1 valid week-end day (i.e., 8 h of wear-time) were retained for the analyses. The Evenson cut points were used to assess the time spent in each physical activity intensity level: sedentary, low, moderate and vigorous. Complementary, diaries were provided to register the activities performed when the accelerometer was removed; these activities were added to the measures provided by the accelerometers. In addition, age-specific self-reported questionnaires (ToyBox and FPAQ) were completed to provide contextual information about the type of activities performed. Due to financial constraints, physical activity in adults (18–64 years) was assessed and described through the self-reported International Physical Activity Questionnaire (IPAQ long version) only.

**Conclusion:**

Data were collected in the context of the BNFCS2014 to provide a comprehensive picture of the physical activity and sedentary behavior in the Belgian population, with a special focus on children (3–9 years) and adolescents (10–17 years). Levels of physical activity and sedentary behavior can be compared to international guidelines and analyzed according to several background variables, such as age, gender, Body Mass Index, education level and region. Such results are aimed to underpin future policies in the field of physical activity.

## Background

Physical activity can be defined as “any bodily movement produced by skeletal muscles which results in energy expenditure above resting level” [1]. Regular moderate intensity physical activity is associated with a reduced risk of cardiovascular diseases, colon and breast cancer, osteoporosis, type 2 diabetes, obesity, high blood pressure, depression, stress and anxiety [2]. In children and young people more particularly, appropriate physical activity helps them to develop healthy musculoskeletal tissues and cardiovascular system, to improve their coordination and movement control and to maintain a healthy body weight [3]. In addition, there is strong evidence to indicate a dose–response relationship between physical activity and health benefits: the more active one is, the greater the health benefits would be [4–7].

Furthermore, independently of the level of physical activity, epidemiological studies have shown that spending excessive time in sedentary behaviors may have a negative impact on several health outcomes [8]. Sedentary behavior concerns “activities that do not increase energy expenditure substantially above the resting level”, i.e., with a metabolic equivalent task unit (MET) between 1.0 and 1.5 [9]. Concretely, it includes activities such as sleeping, lying down, watching television or other screen-based activities [9]. Sedentary behavior was found to be associated with all-cause and cardiovascular mortality [8]. In children and adolescents, sedentary activities, and especially watching TV, were associated with the development of obesity and diverse physiological and psychological problems [8, 10, 11].

Both physical activity and sedentary behavior are thus independently related to health outcomes in children, adolescents and adults. In terms of public health, it is therefore important to assess the level of physical activity and sedentary behavior within the Belgian population and to study to what extent the guidelines in this field are met by the population. Physical activity and sedentary behavior can be measured using indirect (e.g., self-reported questionnaires) and direct methods (e.g., accelerometers). Self-reported measurements allow large samples to be studied at a low cost but do not always reflect accurately the activity patterns due to recall and/or reporting biases, the latter being a typical consequence of social desirability [12–14]. In addition, Chinapaw et al. reported that there is currently no physical activity questionnaire for youth with both acceptable reliability and validity [15], which suggests the need for using objective methods in this age group. However, such questionnaires have the asset to provide information about the type of activities performed and the context in which they take place [16, 17].

To overcome the limitations of self-reported methods, accelerometers have been increasingly used over the last decades, especially in children and adolescents [18]. Such devices are unobtrusive, provide unbiased measurements, can store high amounts of data and are convenient to handle. More specifically, accelerometers provide objective information on the total amount, frequency, intensity and duration of physical activity [16, 19, 20]. Unlike questionnaires, however, accelerometers do not provide contextual information about physical activity and sedentary behavior; the cost of such devices can also hinder their use, especially in large-scale studies [16].

The Belgian National Food Consumption Survey 2014 (BNFCS2014) is the first Belgian nationally-representative study reporting the physical activity and sedentary behavior of children and adolescents based on accelerometer data. The purpose of this paper is to describe the rationale and methodology used in the BNFCS2014 to assess physical activity and sedentary behavior in the Belgian population aged between 3 and 64 years.

## Methods

### Study design

Data about physical activity and sedentary behavior in the Belgian population were collected from February 2014 to May 2015 as part of the BNFCS2014. A detailed description of the protocol, objectives, design and methods of the BNFCS2014 is available elsewhere [21]. The study obtained approval of the Ethical Committee (Ghent University) and the Commission for the Protection of Privacy. Before the first home visit, a signed informed consent was obtained from the participants (or the parents/legal guardians).

The BNFCS2014 collected data from a nationally-representative sample (3297 persons) of the population aged 3 to 64 years living in private households in Belgium at the time of the survey. Residents of institutions (e.g., elderly homes, hospitals, prisons), people who did not speak national languages or having a mental disability were excluded. Participation in the BNFCS2014 involved two face-to-face interviews performed by trained dieticians at the respondent’s home. In children (3–9 years), a parent or legal guardian was used as a proxy respondent. The interviews were equally distributed over the seasons. In addition to physical activity, several kinds of data were collected during these interviews: general information (age, gender, household composition), detailed information about eating habits and food consumption (24 h recall and food frequency questionnaire), data about food safety practices and health issues. Anthropometric measurements, including height, weight and waist circumference, were also gathered following a standardized protocol.

Participation in the BNFCS2014 was voluntary; respondents could opt out of any part of the survey at any time. Although great efforts were made to boost the participation to the study and ensure a representative sampling, the participation rate (i.e., the ratio between the number of full participants, and the number of eligible and unresolved participants [22]) was only 37 %, while the cooperation rate (i.e., the ratio between the number of full participants and the number of eligible participants [22]) was 43 %, which is similar to the rate observed during the previous survey of 2004 (42 %) [21].

**Fig. 1:**
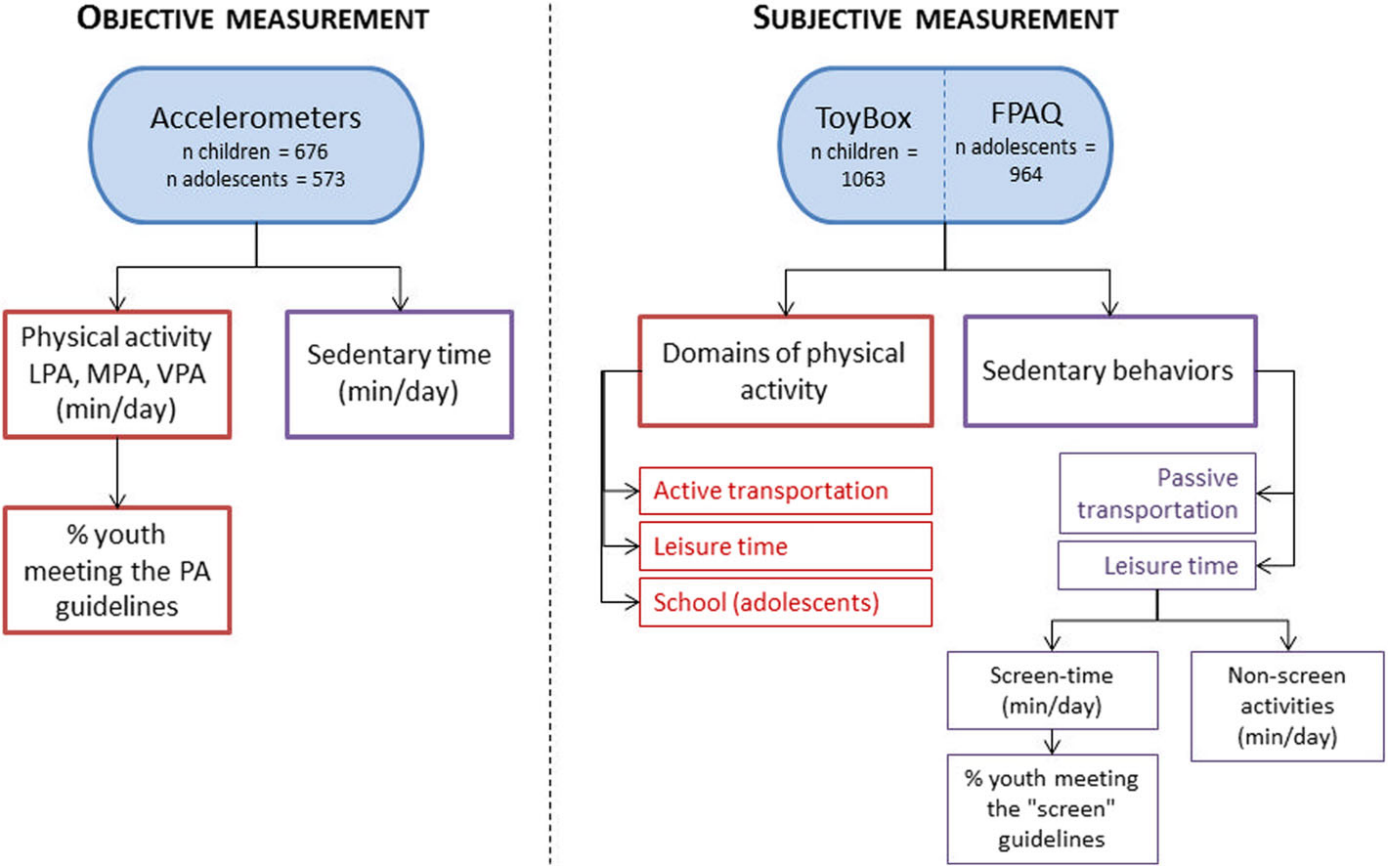
Method used for assessing and describing physical activity and sedentary behavior in children and adolescents. In the context of the Belgian Food Consumption Survey 2014, physical activity and sedentary behavior of Belgian children (3–9 years) and adolescents (10–17 years) were assessed and described based on data from both objective (accelerometers) and subjective (self-reported questionnaires ToyBox and FPAQ) sources. LPA: low physical activity; MPA: moderate physical activity; VPA: vigorous physical activity; PA: physical activity

**Fig. 2:**
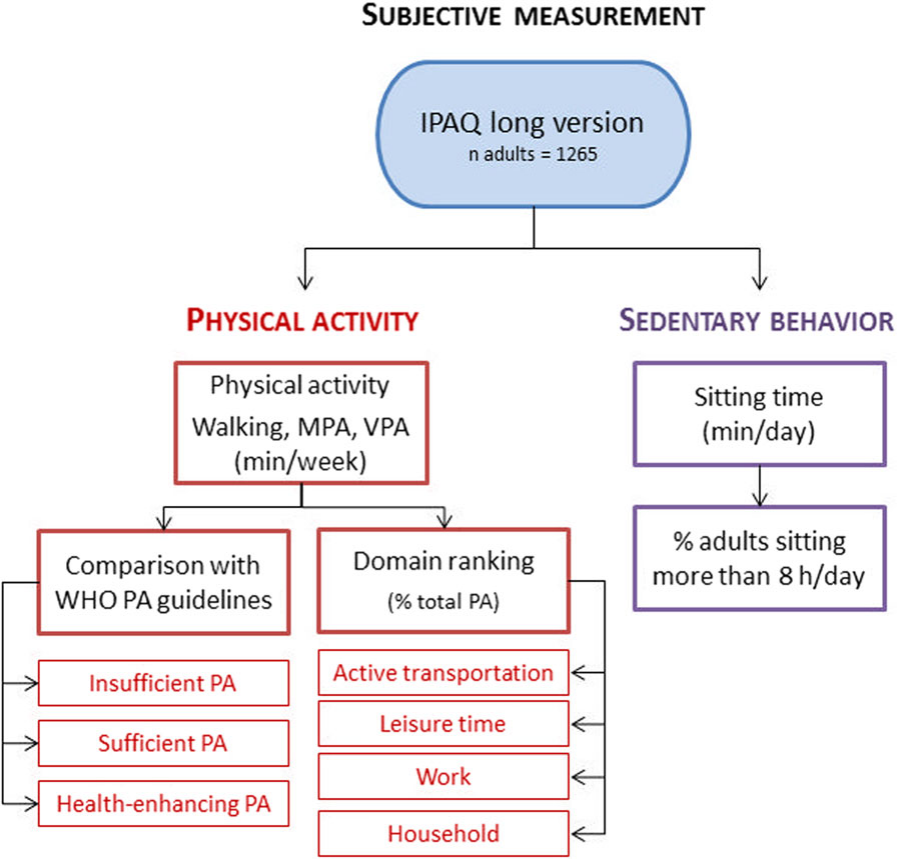
Method used for assessing and describing physical activity and sedentary behavior in adults. In the context of the Belgian Food Consumption Survey 2014, physical activity and sedentary behavior of Belgian adults (18–64 years) were assessed and described based on self-reported data collected through the International Physical Activity Questionnaire long version. MPA: moderate physical activity; VPA: vigorous physical activity; PA: physical activity

Accelerometers and self-reported questionnaires were used in children and adolescents (3–17 years) to objectively assess their level of physical activity and sedentary behavior and to describe the context in which they engage in such behaviors. Due to financial constraints, the assessment among adults (18–64 years) was based on self-reported measurements only. Figures 1 and 2 provide an overview of the methodology and expected outcomes related to the assessment and description of physical activity and sedentary behavior in the Belgian population. The methodological choices performed during the collection, the cleaning and the analysis of the data are described in the following sections.

### Accelerometer measurement of physical activity and sedentary behavior

#### Data collection

In the context of the BNFCS2014, all children (3–9 years) and adolescents (10–17 years) were asked to wear a triaxial accelerometer GT3X+ Actigraph®. This accelerometer model (4.6 × 3.3 × 1.5 cm and 19 g) measures the accelerations caused by bodily movements in three orthogonal planes: vertical, medio-lateral and anterior-posterior. When the device is accelerated, a signal proportional to the intensity of the acceleration is generated. The movement data (called activity counts) are summarized and stored over user-defined time intervals (epochs) [23]. In the present study, an epoch length of 15 s was selected to collect the data. Indeed, previous research has indicated that children tend to engage in short bursts of vigorous activity lasting several seconds (i.e., median of 3 s with 95 % lasting less than 15 s). Whereas 60 s intervals are usually selected in adults, this interval is inappropriate in children and may lead to underestimate the time spent in moderate-to-vigorous physical activity (MVPA) [24, 25].

Actigraph accelerometers have demonstrated acceptable levels of technical reliability and have been shown to be valid in both children and adolescents [23, 26, 27]. However, such findings were based on the uniaxial GT1M model which was widely used in studies assessing physical activity and sedentary behavior [23]. The more recent triaxial GT3X+ model (released in 2009) differs in two main ways from the previous one, i.e., the acceleration measurement in three planes instead of one and the presence of an inclinometer to detect individual posture [28]. Some studies investigated the differences between these two models in terms of results and concluded that both devices assess physical activity similarly, especially for classification of time spent in the intensity categories [28–30].

During the first interview, all children and adolescents were asked to wear a GT3X+ Actigraph® accelerometer over their right hip on an elasticized belt during their waking hours (apart from water activities) for 7 consecutive days. Previous studies investigated the monitor placement and indeed showed that the hip and lower back are the best options [25]. On the other hand, a duration of 7 days was chosen in order to produce acceptable estimates of daily MVPA while taking into account the variation between week and weekend days [25].

The interviewers distributed the accelerometers together with a brochure describing the use of the device. All the information about the use of the accelerometer was also given orally by the interviewer to the children, the adolescents and their parents. In addition, children and adolescents were asked to register in a diary: (i) the time of putting on the accelerometer in the morning and taking it off in the evening; (ii) the time of taking off and putting on the device during the day (when removed more than 5 min); and (iii) the description, the context and the intensity level of the activities conducted when the device was not worn. Such information allows the non-wear activities to be included in the physical activity measurements; including such activities was found to improve the accuracy of the physical activity assessment [31].

The Actilife 6® software was used to initialize the accelerometers before the measurement period, as well as to download the collected data on the interviewer computer afterwards. The accelerometers were initialized to start collecting data at 05:00 AM the day following the first interview. No stop date and time was set in order to monitor seven complete days. Respondents were blind to all data while they wore the accelerometer. The data were downloaded on the interviewer computer and sent to the Belgian Scientific Institute of Public Health (WIV-ISP) – together with the identification number of the respondent – to start the data reduction and cleaning procedure.

#### Data reduction and cleaning

Meterplus® – a Windows-based program developed by researchers from the San Diego State University – was used to prepare and clean the accelerometer data. The assessment of physical activity and sedentary behavior may change substantially depending on the data reduction procedure. A transparent and standardized procedure was therefore used and included the identification of non-wear time, the identification of valid days and the specification of cutoff points. Non-wear time was defined as 20 min or more of consecutive zeroes [32, 33]. Intervals of continuous zero counts shorter than this allowable interruption period were preserved and contributed to the determination of accelerometer wear time. Wear time was then computed by subtracting non-wear time from 24 h [34]. Valid week days were defined as days with wear time of 10 or more hours and valid weekend days as days with wear time of 8 or more hours [31, 35, 36]. Children and adolescents with at least 2 valid weekdays and 1 valid weekend day were retained for further analyses [33, 36]. Because of logistic limitations, it was not possible to offer participants who did not wear the accelerometers sufficiently to have enough valid days, the opportunity to re-wear them.

In this study, physical activity intensity thresholds (i.e., cutoff points based on count values) were used to summarize time spent in four given intensity categories: sedentary behavior, light physical activity (LPA), moderate physical activity (MPA) and vigorous physical activity (VPA). More specifically, the cut-points developed by Evenson et al. were used in the BNFCS2014: < 100 counts per minute (cpm) equals sedentary time, 101–2295 cpm equals LPA, 2296–4011 cpm equals MPA and > 4012 cpm equals VPA [37, 38]. The Evenson cut-points were found to provide acceptable classification accuracy for the 4 levels of physical activity intensity in youth aged 5 years and above [38]. Moreover, count values higher than 15 000 counts per minute were considered to be implausible and put as missing values [26, 39].

#### Non-wear activity diaries

In addition to accelerometer measurements, the participants were instructed to fill in the diaries during seven consecutive days to register all activities during which the accelerometer was removed (except for sleeping). These diaries were collected by the interviewers at the end of the measurement period. They were computerized manually by means of the Blaise® software. About 10.3 % (*n* = 128) of the participants with valid data mentioned one non-water related event for removal of the accelerometer, while 2.2 % (*n* = 27) had two or more of such events. There was a broad spectre of (mainly) play/sport events. The most frequently noted causes for removal were: gym, soccer, dance and basket.

All the activities reported in the diaries were linked with their corresponding MET values based on the Compendium of Energy Expenditure for Youth [40]. The adult compendium [41] was only used for a few activities which were not included in the youth compendium. Non-wear time activities were then classified as sedentary behavior, LPA, MPA or VPA based on the following thresholds: ≤ 1.5 MET equals sedentary behavior, 1.6–2.9 MET equals LPA, 3.0–5.9 equals MPA and ≥ 6.0 equals VPA [40–42]. The registered non-wear time spent in MPA and VPA was multiplied by a correction factor assuming that (i) all the activities during a given time interval are not performed at the same intensity and (ii) reported physical activity time is probably subject to overestimation. According to de Meester et al. [31], the following correction factors were used: 0.5 for leisure activities, 0.8 for organized activities and 0.95 for competition activities [31, 43].

Only children and adolescents having valid accelerometer data and who filled in the diary correctly were included in further analyses. For each valid day, non-wear activity time was summed to the accelerometer-based activity time in order to provide the total sedentary and physical activity time (LPA, MPA and VPA) of this day. As a result of this reduction and cleaning procedure, 778 respondents (38.4 % of the initial sample) were excluded from the analyses for several reasons: refusals, technical problems or invalid data (accelerometer and/or diary data). 676 children (3–9 years) and 573 adolescents (10–17 years) provided valid data and were included in further analyses. Descriptive statistics are summarized in Table 1. Children and adolescents with valid data were more likely to belong to a high educated family, in comparison with children and adolescents excluded from the analyses. No statistically significant differences were found concerning the gender or Body Mass Index (BMI) between young people who were retained for the analyses and those who were excluded.

**Table 1:** Descriptive data for the study population: children (3–9 years) and adolescents (10–17 years), Belgium 2014–2015

Characteristics	Valid data		Excluded	
	Children (*n* = 676)	Adolescents (*n* = 573)	Children (*n* = 387)	Adolescents (*n* = 391)
Gender				
Males	50.7 %	49.2 %	53.0 %	48.1 %
Females	49.3 %	50.8 %	47.0 %	51.9 %
Family education level^a^			*(*P* < 0.0001)	*(*P* = 0.02)
No, primary or secondary school	30.2 %	36.8 %	43.9 %	46.8 %
Higher school short type	34.5 %	29.3 %	25.3 %	25.1 %
Higher school long type	34.0 %	31.4 %	28.4 %	26.3 %
BMI^b^				
Underweight	8.7 %	9.6 %	8.3 %	9.7 %
Normal	77.8 %	72.8 %	73.6 %	70.1 %
Overweight	10.4 %	12.4 %	13.4 %	15.1 %
Obese	3.1 %	4.9 %	4.7 %	4.4 %
Average age (years)	6.68 (±1.96)	14.01 (±2.35)	6.30 (±1.96)**	14.20 (±2.25)
Average number of valid days	6.30 (±1.01)	6.16 (±1.09)	---	---

^a^Family education level (i.e., the highest education level of the parents) was not available for 1.3 % of the children and 2.4 % of the adolescents with valid data, as well as for 2.3 % of the children and 0.8 % of the adolescents excluded

^b^BMI (Body Mass Index) was not available for 0.4 % of the adolescents with valid data and for 0.8 % of the adolescents excluded

*Significantly (*P* < 0.05) different from children/adolescents with valid data, based on chi-square tests; **Significantly (*P* < 0.05) different from children with valid data, based on a *t*-test

#### Outcome measures

Firstly, the average time spent daily at the different intensity levels (sedentary behavior, LPA, MPA and VPA) was computed for each participant and then averaged over the study population to provide estimates of the sedentary time, LPA, MVPA in Belgian children and adolescents.

Secondly, percentages of children and adolescents meeting the physical activity recommendations were computed (Fig. 1). To this end, the World Health Organization (WHO) physical activity guidelines were considered in youth aged between 6 and 17 years: according to these guidelines, young people should accumulate at least 60 min of MVPA daily [1]. Since the WHO does not provide guidelines for preschool children, regional and national recommendations were used for children aged between 3 and 5 years: such guidelines mention that young children should participate in physical activity of any intensity (i.e., light, moderate or vigorous physical activity, LMVPA) for at least 180 min a day [44–46]. The proportion of children and adolescents meeting these guidelines was presented in three ways: (i) the proportion of youth who recorded at least 60 min MVPA (6–17 years) or 180 min LMVPA (3–5 years) each valid day measured; (ii) the proportion of youth who recorded in average at least 60 min MVPA (6–17 years) or 180 min LMVPA (3–5 years) per day; and (iii) the percentage of valid days where ≥ 60 min MVPA (6–17 years) or ≥ 180 min LMVPA were accumulated [47].

### Self-reported information about physical activity and sedentary behavior

#### Data collection

Despite the accurate assessment of the frequency, intensity and duration of physical activity and sedentary behavior by accelerometers, these devices do not provide information about the type of activities performed and the context in which these activities take place [16, 17]. Therefore, self-reported questionnaires were used in addition to accelerometers in children and adolescents (3–17 years) (Fig. 1). Since financial constraints hindered the use of accelerometers in adults (18–64 years), only self-reported questionnaires were used to assess and describe physical activity and sedentary behavior in the Belgian adult population (Fig. 2).

In the BNFCS2014, age-specific physical activity questionnaires were asked to the respondents using a computer-assisted personal interviewing (CAPI) technique. The interviewers asked the questions, while showing the answer categories to the respondents on a card. The answers were directly entered into a portable computer. The CAPI technique, developed in Blaise®, enhances the quality of collected data by including automatic jumps and the ability to identify inconsistent or impossible answers. Moreover, the process of post-collection data entry which constitutes a possible source of error is therefore not needed.

The questionnaire developed in the context of the European ToyBox study (http://www.toybox-study.eu/) and partly based on the work of Burdette et al. [48] was used in **children** (3–9 years). The overall goal of the ToyBox study is to promote healthy lifestyles in children aged 4 to 6 years in order to prevent obesity, through an intervention scheme involving the family and kindergarten stakeholders [49]. The specific questionnaire developed during this project was used in the BNFCS2014. The ToyBox questionnaire was asked to the parents or legal guardians of the children. These questions were focusing on the participation of children in sport clubs, the mode of transportation to get to and from school, the time spent on outside active games and finally the time spent watching television, playing video games or on the computer [50, 51].

The Flemish Physical Activity Questionnaire (FPAQ) was selected to provide contextual information about physical activity and sedentary behavior in **adolescents** (10–17 years). This questionnaire was developed by Philippaerts et al. to describe and assess the physical activity and sedentary behavior of young people in diverse domains, such as school activities, transportation (to go to school and during the leisure time), leisure time and sedentary activities [52]. The computer version of this questionnaire was found to be reliable and valid to assess the physical activity of adolescents aged 12 to 18 years [52]. The paper version of the FPAQ was considered reliable and valid in children from 9 to 11 years, when the questionnaire was filled in with parental assistance [53]. The FPAQ originally developed in Dutch was used in the Flemish part of Belgium; this questionnaire was translated in French by the WIV-ISP to be used in the French part of the country. The questions were asked directly to the adolescents themselves. The FPAQ questions focused on physical activities during and after school time (including the frequency and the duration of these activities) and on sedentary activities, such as watching television, playing on the computer, doing homework, discussing with friends and listening to music.

The long version of the International Physical Activity Questionnaire (IPAQ) was used in order to assess and describe physical activity and sedentary time among the **adults** (18–64 years). The IPAQ (www.ipaq.ki.se), available in a short and a long version, is a tool commonly used to collect self-reported physical activity data and was designed to make comparisons between countries possible [54]. Unlike the short version, the IPAQ long version covers the physical activities performed in four domains: (i) at work; (ii) during the transportation; (iii) during the household tasks; and (iv) during the leisure time [54, 55]. Focusing on the last 7 days, the IPAQ long version aims to assess the frequency (i.e., the number of days; “During the last 7 days, on how many days did you do…”) and average duration per day (i.e., in hours and minutes; “How much time did you usually spend on one of those days doing …”) of walking, moderate and vigorous physical activities related to these domains [54]. It was specified to the participants to report only the activities with a minimum length of ten consecutive minutes [54]. In addition, the last questions concerned the sitting time: “During the last 7 days, how much time did you usually spend sitting on a weekday/weekend day?”.

The IPAQ was extensively validated in the literature [55, 56]. This questionnaire is considered to have “reasonable measurement properties” to monitor the level of physical activity among adults aged between 18 and 65 years old [55]. Generally speaking, the use of the IPAQ is associated with an underestimation of the sedentary time and overestimation of the MVPA duration, thereby also leading to an overestimation of the proportion of persons meeting the physical activity guidelines [57–59]. In addition, IPAQ long version would lead to larger overestimation of the total physical activity in comparison to the short version [55]. Nevertheless, such a questionnaire constitutes an appropriate tool to rank the different domains studied according to their contribution in terms of physical activity [16]. In addition, contextual and domain-specific information provided by this questionnaire is needed to develop effective intervention programs, as different correlates may exist for the different domains [54].

#### Data cleaning and outcome measures in children and adolescents

The procedure used for cleaning the self-reported data in children and adolescents mainly involved checking data consistency (e.g., extreme values), recoding open-field questions and recoding time intervals. For the latter, the lower threshold of the time duration proposed (e.g., 1 to 2 h/day) was systematically considered for the analyses. In children and adolescents, self-reported questionnaires were used to provide contextual information on the specific behaviors they were engaging in while being active or sedentary. Diverse domains in which physical activities can take place were part of the questionnaires and were analyzed: physical activity at school, during the leisure time and during the transportation. Since different questionnaires were asked to children and adolescents, different outcomes were also obtained in these two age groups; caution is therefore required when comparing results of children and adolescents. In total, data of 1063 children and 964 adolescents were considered to produce these outcomes.

In children, data collected allowed the participation in sportive clubs (percentage and average duration in min/week) and the average time spent playing outside (min/day, in week versus weekend days) to be estimated. Many studies have shown that outdoor playing time is positively associated with increased physical activity and improves the development of children motor and social skills [60, 61]. In adolescents the results focused on the duration of physical activity at school (min/week, during and outside the lesson hours) and the participation in sports during the leisure time (percentage and average duration in min/week). The use of “active” modes of transportation (i.e., walking, cycling, skating, scootering) to get to and from school (percentage and average duration in min/week) was studied in both children and adolescents. Indeed, being active during transportation was found to have a positive effect on cardiovascular health and body weight [62, 63]. Physical activity is not limited to leisure time and sport activities but can be practiced in several domains of daily life. Providing data on such domains constitutes therefore a key point to promote the daily practice of physical activity.

Concerning the sedentary behavior, time spent watching TV or videos and time spent playing video games or on a computer were summed to obtain the total screen time (min/day). In addition to their sedentary character, such activities can impact negatively the diet quality, body composition, risk of cardiovascular disease, mental health, quality of sleep and academic performance in youth [64–66]. The results about screen time were therefore used to assess the proportion of children and adolescents having a screen time longer than recommended guidelines, i.e., 1 h per day in pre-school children (3–5 years) and 2 h per day in children and adolescents aged between 6 and 17 years [45, 46, 67]. In children as in adolescents, these indicators were computed for week and weekend days separately.

Sedentary behavior does not include only screen-based activities. Other sedentary activities (e.g., school, “passive” transport, socializing, music) can also contribute substantially to the total sedentary time [68]. Such activities were therefore also investigated in this study: the use of “passive” mode of transportation (i.e., car, bus, tram) by children and adolescents to get to and from school (percentage and average duration in min/week), the time spent by children in playing quietly (min/day) and the time spent by adolescents in diverse types of sedentary activities, such as socializing (e.g., discussing with friends), listening to music, doing homework and reading (min/day). The nature and context of being sedentary is usually poorly understood [68]. A comprehensive picture of the sedentary activities in which children and adolescents are engaged is therefore needed to develop targeted and effective intervention actions to decrease sedentary time in the population.

#### Data cleaning and outcome measures in adults

The procedure used for cleaning and analyzing the data collected in the BNFCS2014 with the IPAQ long version followed mainly the IPAQ protocol adapted by the Ghent University [54, 69]. In summary, these cleaning steps consisted of: (i) computing weekly minutes of physical activity per level (walking, MPA, VPA) in each physical activity domain; (ii) truncating these activity durations to a maximum of 840 min/week for transport-related physical activity, 840 min/week for household physical activity, 840 min/week for recreational physical activity and 600 min/week for professional physical activity; (iii) summing the truncated domain variables to compute the total duration in each physical activity level; and (iv) truncating these total durations to a maximum of 1680 min/week for MPA (including walking) and 780 min/week for VPA [54]. Data were truncated according to the protocol described by Dubuy et al. [69] and Van Holle et al. [54]. In addition, the total duration in each physical activity level was also multiplied by a correction factor of 0.8 to take probable overestimation into account. In the following steps, it was decided to consider walking (whatever the pace) as MPA, since walking has been associated with health benefits [70, 71] and is usually assigned to a MET-value ≥ 3 (even at a moderate pace or for pleasure) [41]. Craig et al. also showed that excluding “slow-paced” walking did not influence the reliability of the data [55]. In total, data of 1264 adults with valid questionnaires were included in further analyses.

In adults, self-reported data about physical activity were used for two purposes: (i) to assess the total time spent in MVPA (including walking) and the proportion of the adult population (18–64 years) meeting the WHO guidelines in this field and (ii) to provide contextual information about domain-specific physical activity. Concerning the quantitative objective, the total MVPA duration in min/week was compared to the WHO guidelines. According to these guidelines, adults aged between 18 and 64 years should accumulate at least 150 min/week of MPA or at least 75 min/week of VPA or an equivalent combination of moderate and vigorous activity [72]. For additional health benefits, adults should engage in 300 min/week of MPA or 150 min/week of VPA or an equivalent combination of moderate and vigorous activity [72]. Based on these thresholds, a categorical variable was created to identify the proportion of adults with (i) insufficient physical activity level; (ii) sufficient physical activity level; or (iii) health-enhancing physical activity level. Such a variable is expected to be overestimated, as also observed in the literature, due to the difficulty to assess the activity intensity, as well as recall and social desirability biases [55, 57, 58, 73]. On the other hand, the WHO thresholds are also questioned: a recent meta-analysis studying the relation between physical activity and chronic diseases suggested that the level of physical activity should be several times higher than the recommended WHO level (150 min/week of MPA) to reduce substantially the risk of chronic diseases [74]. This meta-analysis also emphasizes that more studies are needed quantifying with details the total physical activity to find more precise relative risk estimates for different physical activity domains [74]. From a descriptive point of view, self-reported questionnaires (like the IPAQ long version) constitute however appropriate activity-ranking instruments [16]. In the BNFCS2014, domain-specific information were therefore used to rank the 4 physical activity domains according to their contribution to the total physical activity duration. More specifically, the contribution of each domain to the total MVPA duration (in %) was computed to describe physical activity patterns in the population.

Furthermore, self-reported IPAQ data related to sitting time – during week and weekend days separately – were used to assess sedentary time in the adult population. Even if many epidemiological studies have reported the health hazards of prolonged sitting time [75, 76], there is not yet consensus on the recommended maximum sitting time. In this study, the threshold of 8 h per day was considered as “high sitting time” based on expert consultation. The total sitting time per day (averaged between week and weekend days) was compared to this value to assess the percentage of the adult population sitting more than 8 h per day. Here an underestimation of the adult sitting time is also expected due to recall and social desirability biases [59]. In this regard, objective measurements like accelerometers would provide estimates around 20 % more than the self-reported estimates [77].

### Statistical analyses

Whatever the method of data collection (accelerometers or self-reported questionnaires), all the means and proportions computed were weighted for age, gender and season (using the Belgian population of January 2014 as reference) to provide outputs which are representative of the Belgian population. Outcomes were compared according to the gender, age, family education level, BMI, region and season, after adjustment for age and/or gender. When comparing the results between BMI classes, adjustment for family education level was also taken into account. Statistical analyses were performed using SAS 9.3 and Stata 14. A statistical significance level of 0.05 was used in all analyses.

## Discussion

This paper describes the methodology used in the context of the BNFCS2014 to assess the level of physical activity and sedentary behavior in the Belgian population aged 3 to 64 years. To our best knowledge, this survey will provide for the first time objective accelerometer-based measurement of different physical activity intensities and sedentary time within a large nationally-representative sample of children (3–9 years) and adolescents (10–17 years). In addition, this study will also estimate the proportion of Belgian youth who are sufficiently active according to the current physical activity guidelines, based on objective assessment. Assessing and monitoring physical activity and sedentary behavior in young people is particularly important since active living habits established during childhood are likely to be carried through into adulthood [47, 78]. Using accelerometers has the strength to provide accurate, reliable and practical objective information about the total amount, frequency, intensity and duration of physical activity in daily life, which is especially important in youth where self-reported methods are limited by biased reporting and low validity [15, 18, 79].

Another strength of the current survey is the combination of accelerometer-based measurements with self-reported questionnaires in order to provide important information about the context within which physical activities are performed, about the screen time behaviors and non-screen sedentary activities. The concept of physical activity includes sports but also other kinds of activities: sports are usually planned, organized and repetitive, with the objective of improving or maintaining physical fitness, whereas non-sports activities can be subdivided into different domains such as school, leisure-time and transportation activities [79]. All these kinds of physical activities contribute to the total physical activity and have therefore to be taken into account in targeted and effective public actions aiming to increase the physical activity of the population. On the other hand, understanding sedentary behavior patterns in youth is also essential to inform the development of public health interventions. In the BNFCS2014, screen-time as well as non-screen activities were investigated to understand how and where young people are being sedentary. Such data will notably contribute to assess the percentage of children and adolescents having a screen-time longer than recommended.

Furthermore, an additional strength of the BNFCS2014 is the collection of a wide range of data covering different topics, including eating habits and food consumption, but also background (age, gender, education level, region) and anthropometric data (waist circumference, height, weight). All the outcome variables will consequently be analyzed according to age, gender, family education level, BMI and region. The description of the patterns observed in different subpopulations (males/females, low/high socioeconomic status) is expected to provide evidence for the development of targeted public health actions. These data will also be available for scientific research purposes, e.g. focusing on the physical activity/sedentary behavior in specific age groups (such as pre-school children) or studying the relationships between physical activity patterns and dietary habits. In addition, potential seasonal differences will be investigated. Indeed, previous studies identified an association between the season and the weather, on the one hand, and physical activity level in children, on the other hand [80, 81]. The impact of this parameter should therefore be assessed in Belgium to identify potential obstacles and develop practical guidance accordingly.

Despite these strengths, the method used for assessing physical activity and sedentary behavior in the Belgian population also presents several limits. Firstly, the use of accelerometers in young people raises methodological issues: the selection and potential influence of the epoch length on the results, the selection and impact of the intensity thresholds on the results and the underestimation of some activities. In this study, the epoch length was established to 15 s based on previous research indicating that children tend to engage in short bursts of vigorous activity lasting several seconds [24, 25]. A recent review showed that epochs ranging from 1 to 60 s are found in the literature (the most commonly used being 60 s and 15 s) [82]. Even if strong evidence are not yet available, the epoch length should be considered as an important issue which could impact the MVPA assessment; in addition, some studies have suggested that such an effect would be dependent on the cut-off points selected [82].

Besides the epoch length, the cut-off points have a strong effect on the estimates of MVPA and proportion of youth meeting the guidelines. For example, a review of European studies found that 1 % of children met the guidelines when a MVPA threshold of > 4000 cpm was used, 3 to 9 % of children were considered as sufficiently active with a threshold of > 3000 cpm, and 36 to 87 % of children complied with the recommendations with a threshold of > 2000 cpm [82]. Such discrepancies make the comparison between studies difficult. In the current study, the thresholds developed by Evenson et al. were used [37]. These thresholds have been shown to be valid in youth aged 5 years or older [38]. A lower validity was however found for MVPA in children aged 4 to 6 years [83] and these thresholds have not been validated in children younger than 4 years [47]. MVPA results in the age group 3–5 years will therefore have to be interpreted with caution. This validity issue is nevertheless less problematic regarding the LMVPA (and the percentage of preschool children having at least 180 min LMVPA/day): indeed, LMVPA includes all activities which are not classified as sedentary and the use of the 100 cpm threshold for sedentary time among preschoolers was considered as “the most valid choice” at this moment, as long as more results are not available to refine the assessment of sedentary behavior in young children [84].

Accelerometers also have limitations in their accuracy for measuring physical activity and sedentary behavior. Since they are worn on the hip, they cover inadequately upper body physical activity and physical activity when cycling, thereby leading to a potential underestimation of physical activity in regions (such as Flanders) where cycling is often used for transportation [85]. Accelerometers were removed by the participants during water-based activities, which can also lead to an underestimation of the total physical activity; such a bias was however minimized by using diaries and including non-wear activities. In addition, accelerometers tend to consider motionless-standing activities as sedentary behaviors, while such activities should be rather classified as LPA. This bias can consequently involve an overestimation of the total sedentary time [47]. Non-compliance of young people with these devices constitutes another issue in this field: in the present study, non-wear activities reported in diaries were included. Gift vouchers were offered to the respondents who completed both interviews to boost their participation to the study. Additional underlying strategies, such as supplying information about the cost of accelerometers, individual graphs of activity, regular contact with participants and reminders to wear the accelerometers, could however also have a positive influence on protocol compliance [86].

Secondly, the assessment of physical activity and sedentary behavior in adults was based on self-reported questionnaires only due to financial reasons. As demonstrated in the literature [55, 57–59], this choice will certainly involve an underestimation of the sitting time and proportion of adults sitting more than 8 h per day, as well as an overestimation of the time spent in MVPA and proportion of adults meeting the physical activity WHO guidelines. Furthermore, using a subjective method in adults and an objective one in children and adolescents makes the comparison between these age groups irrelevant and therefore creates an interruption in the assessment of physical activity and sedentary behavior along the life. Based on the data collected in the BNFCS2014, it will consequently not be possible to study the evolution of these variables – in a cross-sectional way – between childhood, adolescence and adulthood. To overcome this limitation, further studies should also objectively measure physical activity and sedentary behavior in adults.

Thirdly, the participation rate of the BNFCS2014 was only 37 % (i.e., the ratio between the number of full participants, and the number of eligible and unresolved participants [22]). Although the results were weighted for age and gender, this low participation rate may introduce bias since it is possible that participants differed from non-participants. For instance, the education level of the sample seems to be quite high in comparison with the actual Belgian population. However, the distribution of the non-response across the sample has to be investigated further.

Finally, the BNFCS2014 is a cross-sectional study which does not allow for inferences of causality or identify changes across time. This is particularly important to keep in mind when comparing outcome measures according to BMI. Potential relationships between physical activity and BMI can indeed be bidirectional: whilst many studies identified the protective effect of regular physical activity against obesity [87], others also showed that an increased adiposity level in children could involve decreased physical activity level [88].

## Conclusion

In conclusion, data were collected as part of the BNFCS2014 to assess the physical activity and sedentary behavior in the Belgian population and to compare these with international guidelines. Using objective and self-reported approaches will provide complementary data on the total physical activity and sedentary time, but also on the context in which the people are engaged in these activities. Such results are needed to boost promotional efforts and to develop targeted and effective public health recommendations promoting physical activity while reducing sedentary behavior. These data are also intended for use in scientific research, e.g., about the determinants of physical activity and sedentary behavior, the time periods when children are most likely to be sedentary/least likely to be physically active, or the relationships between physical/sedentary activities and specific dietary habits.

## Abbreviations

BMI: Body mass index
BNFCS2014: Belgian National Food Consumption Survey 2014
cpm: Counts per minute
FPAQ: Flemish physical activity questionnaire
IPAQ: International physical activity questionnaire
LMVPA: Low, moderate and vigorous physical activity
LPA: Low physical activity
MET: Metabolic equivalent of task
MPA: Moderate physical activity
MVPA: Moderate-to-vigorous physical activity
VPA: Vigorous physical activity
WHO: World Health Organization
WIV-ISP: Belgian scientific institute of public health
